# Association between antidepressant use during pregnancy and autism spectrum disorder in children: a retrospective cohort study based on Japanese claims data

**DOI:** 10.1186/s40748-018-0096-y

**Published:** 2019-01-10

**Authors:** Madoka Yamamoto-Sasaki, Satomi Yoshida, Masato Takeuchi, Sachiko Tanaka-Mizuno, Yusuke Ogawa, Toshiaki A. Furukawa, Koji Kawakami

**Affiliations:** 1 Department of Pharmacoepidemiology, Graduate School of Medicine and Public Health, Kyoto University, Yoshidakonoe-cho Sakyo-ku, Kyoto, 606-8501 Japan; 2 Department of Medical Statistics, Shiga University of Medical Science, Setatsukinowacho Otsu, Shiga, 520-2121 Japan; 3 Department of Healthcare Epidemiology, Graduate School of Medicine and Public Health, Kyoto University, Yoshidakonoe-cho Sakyo-ku, Kyoto, 606-8501 Japan; 4 Department of Health Promotion and Human Behavior, Graduate School of Medicine and Public Health, Kyoto University, Yoshidakonoe-cho Sakyo-ku, Kyoto, 606-8501 Japan

**Keywords:** Maternal depression, Antidepressant, Pregnancy, Autism spectrum disorder

## Abstract

**Background:**

Studies using data from Western countries have raised concerns that treating pregnant women with antidepressants may increase the risk of autism spectrum disorders (ASDs) in their offspring. However, to date, the studies are inconclusive. We therefore examined the association between antidepressant use and ASD using claims data collected in Japan.

**Methods:**

This retrospective cohort study was based on claims data from mothers and their children from January 2005 to July 2014, obtained from the Japan Medical Data Center. The information from mothers and children was linked using the family identification code. Information on antidepressant prescriptions during pregnancy was extracted from the database. To collect information on ASD, children for whom data were available 24 months or more after birth were followed up from birth through July 2014 or up until their withdrawal from the database. To ensure appropriate diagnosis of ASD, mother-child pairs where the children’s data did not cover the 24 months after birth or pairs where children had a diagnosis of ASD within only 23 months after birth were excluded from the study cohort. We used logistic regression analyses to evaluate the association between antidepressant use during pregnancy and the children’s ASD diagnosis. All statistical analyses were performed using IBM SPSS (Statistical Package for the Social Sciences) Statistics ver. 21.0.

**Results:**

Of the 53,864 eligible mother-child pairs, 26,925 met the study criteria. Crude analysis showed that the ASD prevalence in children was significantly higher with any antidepressant use than with non-use (odds ratio [OR], 2.32; 95% confidence interval [CI], 1.08, 4.95). However, when the analysis was adjusted for the confounding effect of maternal depression during pregnancy, statistical significance was lost (OR, 0.76; CI, 0.27, 2.18).

**Conclusions:**

After adjustment for confounders, we found no significant association between antidepressant use during pregnancy and ASD in children in Japan. This result provides additional evidence to support the idea that antidepressant use during pregnancy itself is not associated with an increase in ASD in children. In addition, this represents the first evidence based on Asian data.

## Introduction

Depression is a common mental disease during pregnancy [1]. Untreated prenatal depression increases the risk of pregnancy-related complications, such as gestational diabetes, poor obstetric outcomes, and adverse consequences to the child’s development [2, 3]. In recent years, antidepressant use during pregnancy has increased to approximately 2–7% in Western countries [4, 5]. However, this practice has raised concerns of a possible increased risk of autism spectrum disorder (ASD) in the children, as reported in some studies [6–9]. On the other hand, some studies did not show any significant association when confounders were taken into consideration [10–15]. Therefore, it remains to be clarified whether the use of antidepressants during pregnancy increases the risk for ASD in children.

All previous studies on this issue have used only subjects in Western countries; no such study has to date been conducted in Asian countries. In addition, it is known that antidepressant use during pregnancy in Japan is lower than in Western countries. Thus, using Japanese subjects we sought to evaluate whether the use of antidepressants during pregnancy increases the risk for ASD in the offspring. In this study, we describe a retrospective cohort study of 26,925 mother-child pairs in which we examined the prevalence of ASD in the children of mothers who used or did not use antidepressants during pregnancy, based on claims data from Japan.

## Methods

### Study design and data source

This study was a retrospective cohort study of 53,864 mother-child pairs. We obtained our data from an administrative claims database maintained by the Japan Medical Data Center (JMDC, Tokyo, Japan). The database consists of insurance eligibility data and includes medical and pharmacy claims. Information in the database included gender, year and month of birth, medical services provided or drugs prescribed, the date of diagnosis, inpatient or outpatient status, and the size and specialty of the medical facilities used. The data were labelled by unique personal identification codes and family codes.

### Subjects

We extracted mother-child pairs from the database where data for the 11 months prior to delivery were available for the mother, from January 2005 to July 2014 (mothers, 47,388; children, 53,864). We linked mother and child information using the family identification code. We selected pairs where the children’s data were available for at least 24 months after birth, and excluded pairs where the children’s data did not cover the 24 months after birth or pairs where the children had a diagnosis of ASD within only 23 months of birth to ensure an appropriate diagnosis of ASD.

### Exposure

To evaluate exposure, information on the antidepressants prescribed to the mother during pregnancy was collected. An antidepressant was defined according to the Anatomical Therapeutic Chemical (ATC) classification used by the database (i.e., ATC N06A). The pregnancy period of mothers was taken as 9 months calculated from the month preceding the birth month of the child, and the 9 months were divided into 3 periods of 3 months, namely first trimester, second trimester, and third trimester. If the mother had been prescribed antidepressants during any of these periods, the child was included in the group of those exposed during the fetal period regardless of the duration of antidepressant use. Antidepressant use was classified by pharmacological type into three categories: selective serotonin reuptake inhibitors (SSRIs), serotonin-norepinephrine reuptake inhibitors (SNRIs), and all other types.

### Outcomes

The incidence of ASD was determined from the diagnoses of ASD as coded by the International Classification of Diseases, 10th revision (ICD-10). Children were considered to have ASD if they had a diagnostic code of ICD F840, F841, F845, F848, or F849. However, as already mentioned, children receiving a diagnosis of ASD within 23 months of birth only were excluded to ensure an appropriate diagnosis of ASD. We followed up the selected children from birth through July 2014 or their withdrawal from the database.

### Statistical analysis

We used maternal age at birth, gender of the child, and maternal depression diagnosis (ICD F31, F32, or F33) during pregnancy as baseline characteristics, and compared the baseline characteristics between the exposed and unexposed groups.

For outcome analyses, we used logistic regression analyses to estimate the odds ratios (ORs) and their 95% confidence intervals (CIs). First, we conducted a crude comparison of exposed children with unexposed children. Next, we performed two analyses adjusted by covariates. In the first analysis, the maternal age at birth and the gender of the child were used as covariates (Model 1). In the second analysis, maternal age at birth, gender of the child, and maternal depression diagnosis during pregnancy were used as covariates (Model 2). Finally, we conducted a restricted analysis limited to children of mothers with a depression diagnosis during pregnancy.

All statistical analyses were performed using IBM SPSS (Statistical Package for the Social Sciences) Statistics ver. 21.0 (International Business Machines Corporation, Armonk, New York, USA).

## Results

**Fig. 1:**
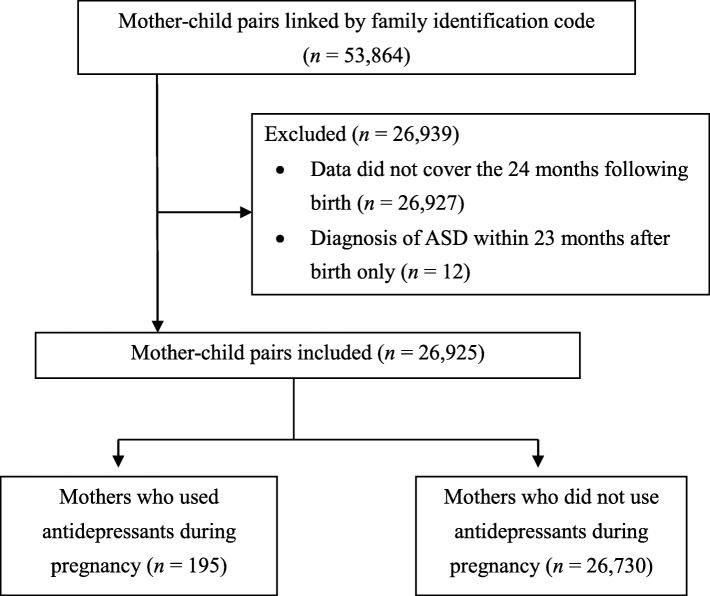
Flowchart of the process of identifying mother-child pairs for inclusion in the study. Abbreviations: ASD, autism spectrum disorder

Of a total of 53,864 pairs found in the database, 26,925 met the recruitment criteria as outlined in Fig. 1 and were retained in the cohort. Of these, 195 mothers (0.7%) used antidepressants during pregnancy, and 26,730 mothers (99.3%) did not.

### Baseline characteristics of the cohort

Table 1 shows the baseline characteristics of the subjects. The mean maternal age at birth of antidepressant users and non-users was 32.1 and 31.4 years, respectively. Mothers who received a depression diagnosis during pregnancy comprised 81.5% of antidepressant users. The percentage of children of antidepressant users and non-users of male gender was 49.7 and 51.3%, respectively.

**Table 1:** Baseline characteristics of antidepressant users and non-users

	Antidepressant use during pregnancy	
	User (*n* = 195)	Non-user (*n* = 26,730)
Maternal age at birth^a)^		
mean (SD), y	32.1 (4.57)*	31.4 (4.39)
< 20, *n*, (%)	0 (0.0)	17 (0.1)
20–29, *n*, (%)	59 (30.3)	9001 (33.7)
30–39, *n*, (%)	124 (63.6)	16,838 (63.0)
≧40, *n*, (%)	12 (6.2)	874 (3.3)
Diagnosed as depressed during pregnancy^b)^, *n*, (%)	159 (81.5)**	120 (0.4)
Child gender ratio (% male)	49.7	51.3

Abbreviations: SD, standard deviation

^a)^*t*-test; *, *P* < 0.05

^b)^*χ*^2^ test; **, *P* < 0.01

The sample included 430 children diagnosed with ASD (1.6%). Boys were more likely to have a diagnosis of ASD than girls, with a ratio of 3:1 (335 [1.2%] vs. 95 [0.4%]).

### Details of antidepressant use

Table 2 shows the type of antidepressant used and the trimester of pregnancy of antidepressant use. The numbers of mothers prescribed SSRIs, SNRIs, and/or antidepressants of other classes were 152 (77.9%), 10 (5.6%), and 50 (25.6%), respectively. The numbers of mothers prescribed any of the above-mentioned three drugs in the first, second, and/or third trimester were 187 (95.9%), 72 (36.9%), and 58 (29.7%), respectively.

**Table 2:** Type of antidepressant used and prevalence of antidepressant use in each trimester

	Prevalence of antidepressant use in each trimester^a)^, *n* (%)			
	All	First	Second	Third
Type of antidepressant				
All types	195	187	72	58
SSRIs	152 (77.9)	145 (77.5)	62 (86.1)	48 (82.8)
SNRIs	10 (5.6)	10 (5.3)	3 (4.2)	3 (5.2)
Other	50 (25.6)	49 (26.2)	15 (20.8)	13 (22.4)

Abbreviations: *SSRI* selective serotonin reuptake inhibitors, *SNRIs* serotonin-norepinephrine reuptake inhibitors

^a)^The 9 months prior to the birth month of the child were divided into 3 × 3-month intervals, namely the first, second, and third trimesters.

### Association between antidepressant use and ASD

Table 3 shows an association between any antidepressant use and any ASD in the children. In the crude and Model 1 analyses, ASD risk was significantly higher with any antidepressant use than in the non-use group (crude, OR, 2.32, CI, 1.08, 4.95; Model 1, OR, 2.37, CI, 1.10, 5.09). However, in the Model 2 analysis, which included maternal depression diagnosis during pregnancy as a covariate, statistical significance was lost (OR, 0.76; CI, 0.27, 2.18).

**Table 3:** Odds ratios for ASD diagnosis in the children of mothers using/not using antidepressants during pregnancy

Antidepressant exposure	All children	Children with ASD	Age at ASD diagnosis^a)^	Crude OR		Model 1 OR^b)^		Model 2 OR^c)^	
	*n*	*n* (%)	average (SD), month	OR (95% CI)	*P* value	OR (95% CI)	*P* value	OR (95% CI)	*P* value
Non-user	26,730	423 (1.58)	39.1 (14.8)	1 (Reference)	–	1 (Reference)	–	1 (Reference)	–
User	195	7 (3.59)	41.3 (18.5)	2.32 (1.08, 4.95)	0.03	2.37 (1.10, 5.09)	0.03	0.76 (0.27, 2.18)	0.61

Abbreviations: OR, odds ratio; CI, confidence interval

^a)^Age at which the child was first diagnosed with ASD.

^b)^Adjusted for maternal age at birth and gender of the child.

^c)^Adjusted for maternal age at birth, gender of the child, and maternal depression diagnosis during pregnancy (Disorders F31, F32, or F33; International Classification of Disorders).

Table 4 shows the results of the subgroup analysis restricted to only children of mothers with a depression diagnosis during pregnancy. There was no significant difference in the ASD risk between the antidepressant use and non-use groups (OR, 0.42; CI, 0.13, 1.37).

**Table 4:** Odds ratios for ASD diagnosis in the children of mothers with a diagnosis of depression

Antidepressant exposure	All children	Children with ASD	Adjusted OR^a)^	
	*n*	*n* (%)	OR (95% CI)	*P* value
Non-user	120	8 (6.67)	1 (Reference)	–
User	159	5 (3.14)	0.42 (0.13, 1.37)	0.15

Abbreviations: *OR* odds ratio, *CI* confidence interval

^a)^Adjusted for maternal age at birth and gender of the child.

## Discussion

In this cohort study, children of mothers using antidepressants during pregnancy were found to be at higher risk of ASD compared with children of non-users, before adjustment for confounders. However, after adjustment for confounders, including maternal depression during pregnancy, the excess risk of ASD was not statistically significant. In addition, in the subgroup analysis restricted to only children of mothers with a depression diagnosis during pregnancy, no increased risk for ASD was found. These results mean show that antidepressant use itself during pregnancy is not associated with an increased risk for ASD in the offspring.

Previous studies examining the association between antidepressant use during pregnancy and ASD in children have produced conflicting results, presumably due to differences in study design and/or study conditions, such as differences in confounders. In population-based or retrospective cohort studies using data from the Netherlands, USA, Sweden, or Canada, the excess risk of ASD in children associated with maternal exposure to antidepressants during pregnancy was not significant after adjustment for maternal psychiatric conditions [10–12], or after further analysis, such as using a sibling or subgroup analysis restricted to children of mothers with a diagnosis of affective disorders [13–15]. The results of these studies are consistent with the present results. However, population-based case-control studies using data from the Netherlands, USA, or Sweden reported an additional risk of ASD in children due to maternal exposure to antidepressants during pregnancy [6–8]. However, case-control studies often overestimate risk, and antidepressant treatment might indicate other exposures that lead to increased risk for ASD [5].

All previous studies on this issue have used data from Western countries; none until now has used data from an Asian country. Our study is the first to use Asian data, and the prevalence of ASD in the children was found to be approximately 1.6% (430/26,925), similar to the current estimates for developed countries of approximately 1.5% [16].

The similar prevalence of ASD in the children despite the low antidepressant use during pregnancy seen in our study suggests that there is no association between antidepressant use during pregnancy and ASD in children. In our study, the percentage of mothers diagnosed with depression during pregnancy was approximately 1.0% (279/26,925), much lower than the percentage in Western countries (approximately 10–15%) [17, 18]. In addition, we found a 0.7% (195/26,925) usage of antidepressants during pregnancy in the Japanese data, much lower than in Western countries where it is approximately 2–7% [4, 5]. In a study from Hong Kong, antidepressant use was found in approximately 0.7% of pregnant women [19], the same as in our study. In that report, the relatively lower percentage was ascribed to the conservative approach of local medical practice. The lower percentage of antidepressant use in our study might also reflect the conservative social practices generally prevailing in Japan. However, our study agrees with previous reports from Western countries [5, 20] in that the most commonly used type of antidepressant during pregnancy was an SSRI.

ASD has a multi-factorial etiology [21], and the presence of a genetic contribution is strongly supported by twin and family studies [22, 23]. ASD is believed to have multiple genetic variants. Moreover, the etiology and common variant alleles contributing to polygenic risk in ASD are thought to be shared, at least in part, with other neurodevelopmental or psychiatric disorders [16]. Notably, the incidence of depression was found to be higher in the first-degree relatives of young children with ASD [24]. Thus, it is plausible that a genetic etiology of maternal depression links it to the genetic etiology of ASD in the child.

Our study using Japanese claims data showed that antidepressant use during pregnancy leads to no increased risk for ASD in children vs. non-use after accounting for confounding factors, including depression in the mother. The results support the conclusion that antidepressant use during pregnancy is itself not associated with an increase in ASD in children. Medical treatment of pregnant women with antidepressants improves both the mental condition of the woman and the development of the child. However, concerns about possible antidepressant influences on development, including influences on ASD in children, persist. Clarification of the risks and benefits of using antidepressants during pregnancy is helpful for patients and clinicians, and our study will aid this. Further studies are necessary before a determination of the association between antidepressant use during pregnancy and ASD in children can be made.

### Strengths

Our study used a large-scale database consisting of data from families who joined company insurance programs, and approximately 27,000 mother-child pairs were included in the study. Our results therefore have generalizability. Furthermore, to our knowledge, ours is the first retrospective cohort study using Asian data to explore the association between antidepressant use during pregnancy and ASD in children.

### Limitations

This study has several limitations. First, the data were obtained from an administrative claims database that did not include some important information, such as information on socioeconomic status, which is considered to be related to maternal depression. Second, maternal depression was found in approximately 1% of pregnant women, and antidepressant use was approximately 0.7%. These are lower numbers than in the West, as reported in the literature. It is unknown whether these low percentages are due to database characteristics or regional differences. Due to the relatively small number of women in our sample who used antidepressants during pregnancy, we could not secure a sample size adequate to support analysis of the effects of antidepressant type or trimester of pregnancy. Further assessment using a cohort of a size adequate for these analyses should be conducted. Third, we included no data dated prior to the pregnancy of the mother. Therefore, we could not consider information on the presence of depression or the prescription of antidepressants prior to the pregnancy of the mother. In addition, we lacked information that would enable us to consider genetic factors contributed by the father.

## Conclusions

In a Japanese sample, after adjustment for confounders, we found no significant association between the use of antidepressants during pregnancy and ASD in the offspring. The result provides additional evidence to support the idea that antidepressant use during pregnancy is not associated with an increased risk for ASD in children. In addition, the result is the first evidence based on Asian data regarding this association. Clarification of the risks and benefits of using antidepressants during pregnancy is helpful for patients and clinicians, and our study will aid this. However, some previous studies have shown conflicting results to our study, and the concerns that the use of antidepressants during pregnancy increases the risk for ASD in children persist for both patients and clinicians. Further studies are necessary before an association between antidepressant use during pregnancy and ASD in children can be determined.

## Abbreviations

95% CI: 95% confidence interval
ASDs: Autism spectrum disorders
ATC: Anatomical Therapeutic Chemical
IBM SPSS Statistics: International Business Machines Corporation Statistical Package for the Social Sciences Statistics
ICD-10: International Classification of Diseases, 10th revision
JMDC: Japan Medical Data Center
OR: Odds ratio
SNRIs: Serotonin-norepinephrine reuptake inhibitors
SSRIs: Selective serotonin reuptake inhibitors
